# The importance of reliable information exchange in emergency practices: a misunderstanding that was uncovered before it was too late

**DOI:** 10.1186/s12910-015-0038-4

**Published:** 2015-07-07

**Authors:** Halvor Nordby

**Affiliations:** The University College of Lillehammer, PO Box 952, 2604 Lillehammer, Norway

**Keywords:** Cardiac arrest, Prehospital work, Resuscitation, Advance directives, Clinical judgment, Rule-based ethics, Virtue ethics

## Abstract

**Background:**

Many medical emergency practices are regulated by written procedures that normally provide reliable guidelines for action. In some cases, however, the consequences of following rule-based instructions can have unintended negative consequences. The article discusses a case - described on a type level - where the consequences of following a rule formulation could have been fatal.

**Case presentation:**

A weak and elderly patient has cardiac arrest, and a Do Not Resuscitate (DNR) clause is written in the patient’s medical record. Paramedics at the scene cannot see that the patient’s general appearance match conditions which would indicate the DNR clause, so they start cardiopulmonary resuscitation (CPR), and the patient survives. This turns out to be a crucial decision. The DNR clause is from an earlier bout with serious disease from which the patient has recovered, against all odds, and someone has forgotten to remove the clause from the medical record.

**Analysis:**

In order to be able to interpret the validity of written guidelines, paramedics and other health workers need to develop personal skills that transcend the ability simply to follow written instructions. Within traditional virtue ethics, personal judgment is conceived of as crucial for being able to make ‘good’ autonomous decisions. Virtue ethical analyses, decision-making abilities and non-technical communication skills are important as conceptual tools when health workers need to make difficult clinical decisions.

**Conclusion:**

The case study accentuates the significance of prudent judgment in medical practice. In the case described, the consequence of trusting the written advance directive could have been fatal, but the point is general: for the purpose of achieving excellent organizational performance, it is insufficient for health workers to rely uncritically on rules and procedures. Even the clearest rule formulations must be interpreted contextually in order to determine ethically correct behavior and avoid potential negative consequences that are not in the patient’s best interests.

## Background

The case discussed in this article has a general form, but it is based on situation described by a participant in an advanced study program for paramedics working in the Norwegian national ambulance services. Other students in the program have also experienced situations similar in kind to that described below, and they all characterized them as some of the worst cases of misinterpretation they had ever encountered in their professional practice.

While these specific situations had many idiosyncratic aspects, they clearly raised the same issues about health communication and clinical judgment, relevant in a range of situations. The learning potential is obvious: Learning how exchange of information sometimes fails can give health workers a better understanding of how they can achieve reliable communication [1, 2].

An initiative was made to write an article about the kind of misunderstanding the paramedic student described, so that lessons to be learned from his narrative could reach a broader audience than those who were directly involved. The paramedic was asked to give his permission to do this, and he replied that he thought it was a good idea. As he said, ‘what happened can be food for thought not only for paramedics, but for all health workers who are involved in a crossfire of organizational communication’.

The next section of the article presents the case study as described by the paramedic. Section three uses the case to argue for two important points: (1) Written procedures for patient work must be interpreted contextually – rule formulations should not be thought of as *obvious* truths with straightforward conditions for applications. (2) In order to avoid potentially negative consequences for patients, health workers need to secure reliable information exchange and exercise good clinical judgment. The final section of the article discusses these points in the light of theoretical virtue ethics.

## Case presentation

The following description is formulated in completely anonymous terms. It is based on a real case, but potentially identifiable details have been removed. The case cannot be connected to a specific place or circumstances and cannot be traced to the identity of any individual patient.

An ambulance unit with two paramedics was summoned to a patient who was reported to have chest pains consistent with heart attack. The patient was living in a care home for the elderly, and staff nurses had made the call for emergency medical assistance. On-line information, which was sent from the medical emergency control centre to the ambulance while it was on its way to the patient, made it clear that the patient was old and had a terminal disease.

When the paramedics arrived, a nurse came running and told them to hurry because the patient had stopped breathing ‘just a couple of minutes ago.’ After being ushered into the patient’s room, the paramedics found an old patient with cardiac arrest. A nurse was doing vague attempts of cardiac compressions, another was checking for signs of a pulse, and they both expressed relief when the paramedics arrived. While one paramedic prepared equipment and the other took over the cardiac compressions and gave instructions to the nurses, the nurse who had showed them into the room told them that the patient’s medical record had a ‘Do Not Resuscitate’ (DNR) clause. As the paramedics knew, this is meant to make it clear that the patient should not be resuscitated if suffering from cardiac arrest.

The nurse showed the paramedics the DNR clause by holding up a page from the record where the clause was handwritten. The nurse claimed not to know the patient very well, but she knew that the patient had a serious terminal disease, and she had found the DNR clause in the patient’s record while waiting for the paramedics to arrive.

The paramedics had now established that the patient did not have a pulse. However, the patient’s skin was still warm, so it was not evident that resuscitation could not bring the patient back to life. Moreover, the paramedics were struck by the patient’s general appearance; it did not suggest that the patient was in a terminal condition caused by the patient’s disease. For the paramedics, five seconds of doubt was all they needed. They thought it was better to be on the safe side and started CPR rather than relying on the validity of the DNR clause.

The patient survived, and the paramedics later learned what had happened: the DNR clause was from an earlier bout with serious disease. At that time, the patient was so weak that the patient, together with close relatives and the patient’s doctor, had deliberated and agreed to insert the clause in the medical record. However, the patient did not have a cardiac arrest, and eventually, against all odds, recovered from the serious disease.

After being free of symptoms for many years, the disease was re-diagnosed. This time, however, it had not developed very much, so when the patient suffered a cardiac arrest, the disease was less threatening than the first time. All in all, the DNR clause was far from justified.

As said above, this case description is based on a particular narrative presented by a paramedic student in a further education program, but other students have described similar forms of misunderstandings. Their narratives have many idiosyncratic aspects, but they all fall under the general type description above – they all involve misinterpretation of DNR clauses in patients’ medical records.

Given the general description of the case described, it is not relevant to seek informed consent from any persons involved. Furthermore, as no other health workers are mentioned in the description, it is also detached from recognizable institutions and health personnel.

## Discussion

The fact that the case description cannot be traced to a specific situation is not crucial from a pedagogical perspective. The important point is that the description is a realistic example of how information exchange can fail. Through analyzing illuminating examples such as this, health workers can improve their awareness not only of why clinical judgment is important, but also why it is imperative to achieve effective communication [3, 4].

The most striking aspect of the case is that the consequences for the patient would have been fatal if the paramedics on the scene had not exercised good clinical judgment. Ordinarily, a directive like a DNR clause provides reliable guidance for action. A DNR clause is an example of an advance directive. This is, as Capron ([5], p. 261) observes, “a statement made in advance of an illness about the type and extent of treatment one would want, on the assumption that one may be incapable of participating in decision-making when the need arises.”

A DNR clause is normally grounded in a document stating the reasons why the patient should not receive CPR in case of cardiac arrest.^1^ The document is ordinarily based on a decision process involving the patient, the patient’s responsible physician and relatives of the patient. It is imperative that the document is signed by the patient and represents his or her wishes as long as the patient is autonomous and capable of expressing informed preferences. As Pietsch and Braun ([6], p.45) observe:

Professionals assisting an individual with end of life decision making, including attorneys and health care providers, have professional responsibilities to determine the mental status of the individual to ensure that decisions are informed, voluntary, and not made under the undue influence of another person.

If the patient is unconscious or has lost the ability to express autonomous wishes, relatives are normally entitled to make decisions about resuscitation and DNR clauses.^2^ If relatives wish to insert a DNR clause in the patients’ record, there must be convincing reasons why the wishes of the relatives represent the best interests of the patient [7]. In future interaction with the patient, it is imperative that health workers can be confident that the DNR clause is valid - that the patient would have accepted the DNR clause if the patient had been capable of expressing autonomous preferences [8, 9].

Nevertheless, the case above illustrates that there are always human factors involved in implementing procedural directives like DNR clauses. A DNA clause is an example of a system-based rule – a written instructive that health workers should conform to – and the interpretation of any rule can, in principle, be experienced incorrect in the sense that actions made in accordance with the rule can be conceived as ethically wrong [10]. Furthermore, instructive rules can be mistakenly applied, as this almost happened in the given case. This means that rule formulations must be interpreted contextually. Written instructions such as a DNR clause should not be thought of as *obvious* truths with straightforward conditions for application. As the case illustrates, this point is especially important when following a potentially incorrect rule formulation can have serious and immediate negative consequences for patients.

Obviously, this does not mean that health workers are entitled to overrule instructional directives whenever they think it is appropriate. The fact that it can be correct to set aside a written instructional directive in some exceptional circumstances, does not mean that everyone should trust their personal judgments if they conflict with guidelines. The point that the case illustrates is the much less radical point that instructional directives can be overruled if there are contextual reasons for thinking that they are based on a misunderstanding, and if acting in according with unjustified directives can have negative consequences for the patient.

This point falls under a more general principle: an instructional directive can only be valid if the patient is in the state of ill health described in the directive. As Pietsch and Braun ([6], p.44) observe, “under normal circumstances, for the advance directive to be followed, a physician must first certify that the patient is in a certain condition or state of incapacity”. In prehospital work, when physicians are not present in the patient encounter, paramedics have in principle the same professional duty to consider the patient’s state off ill health. However, it takes time to assess the patient parameters thoroughly, and as Pietsch and Braun ([6], p.44) points out, “these types of determinations are not easily made in emergency [work]”. Thus, if uncertainty about a patient’s condition means that there is uncertainty about the application of an advance directive that can have unjustified negative consequences for the patient, it is better to be on the safe side and follow normal procedures for life sustaining treatment.

### Professional autonomy

The importance of individual interpretation in acute medical practice is normally acknowledged in official guidelines for how procedures should be applied [8, 9, 11, 12]. Paramedics are to some extent entitled to make autonomous decisions, but this depends on their formal level of competence and the structural organization of the ambulance services in which they work.^3^ In Norway it has been decided that where possible, decisions to terminate CPR in pre-hospital settings should be made by consulting physicians who are formally responsible for patients. Paramedics have medical operative manuals (MOM) where this is stated as a requirement. These manuals also clarify, to some extent, how paramedics should make their own judgments about CPR. It is emphasized that they must consider the best interests of the patient and make well-founded ethical judgments.^4^

However, these general competence skills are not connected to procedures, and there are no guidelines about situations of the above kind – when paramedics are in doubt about the soundness of a DNR clause. One obvious reason why there are no detailed rules about this is that if the MOM had included very many situation-specific rules, it would have been difficult to learn and remember all of them. The manual would have been a ‘jungle of rules’ that it would be impossible to use efficiently and quickly in real life acute situations. Another reason why specific rules about DNR clauses and CPR are not included in the MOM, is that it is difficult to formulate rules that capture all relevant aspects of difficult situations paramedics can face. Contextual differences make it difficult to formulate strict rules that always give the ‘correct’ answer in a type of situation described on a general level.

There are, at the same time, principles about DNR clauses that all paramedics should conform to. For instance, it is insufficient that relatives of the patient show paramedics a note where the patient states that he or she does not want to be resuscitated. It is difficult to know for sure how autonomous the patient was when writing such a note, and it is possible, at least in principle, that the individual changed his or her mind after writing it [5, 9].

If there is reasonable doubt about the patient’s preferences, then it is better to be on the safe side and do CPR to avoid potential negative consequences that are not in the patient’s best interests. This idea of being ‘on the safe side’ is important. Paramedics and other health workers should not turn this the other way around. They should not apply a procedure rule if there is reason to doubt that it fits the situation they are in, and if applying it can have unwarranted negative consequences for the patient.

In situations of the kind described above, it is hard to see how the paramedics can risk any kind of legal problem as long as they can defend their actions by appealing to their clinical judgment. It is important to remember that the paramedics had to act quickly. There was not much time for deliberation, and if it had turned out that the DNR clause was valid, the paramedics could have stopped CPR once they got more information. Furthermore, they did not violate the principle that the possibility of not starting CPR should be discussed with the most qualified personnel at the scene [9, 12]. They talked about what they should do as best as they could, and chose - as an action-guiding principle - to focus on the idea that CPR should be started when there is a minimal hope of recovery. Thus, their actions were grounded in a basic *prima facie* norm: if there is significant doubt, CPR should be started. This point falls under a more general principle about professional ethics. Like other health workers, paramedics have a professional duty to save lives and improve conditions stemming from disease or injury [11, 12].

However, even when this is the aim, the ‘human factor’ plays a role in interpretation and decision-making [13–16]. The significance of individual reasoning, contextual interpretation and inter-professional consultation fall under a more general principle, namely, that health workers – even those who are not formally responsible for patients – should develop their own views and present them in decisional processes. Health workers should not think of themselves as mechanical pieces in a game and always defend their actions purely by appealing to literal rule following. It is important to remember that it is those who are working in the front line who actually see the patients and their symptoms. They have the ‘clinical eye’ and make direct observations. A consulted doctor on the phone does not have direct *experiential* knowledge of the patient’s condition and the context of the encounter.

#### Theoretical framework

Theoretically, the point about not trusting rules blindly belongs within virtue ethics, a moral tradition that goes back to the philosopher Aristotle and his analysis of what it means to become a competent practitioner [17]. According to virtue ethics, the value of an action depends on the virtues of the person who chooses that action. If a person acts out of good attitudes and relevant competence skills, then his actions are good. If a person has negative motives, or if he is not competent to make informed decisions, his actions are not justified ethically. Davis et al. ([18], p.48) define virtue ethics as a position that presupposes that the “character and integrity of health workers as individual moral agents determine or, at the very least, influence whether ethical problems are identified and how responses are developed to such problems in patient care”. Virtue theorists hold that

… [c]haracter and virtue, often considered to be too subjective, have a place in today’s professional health care ethics… Descriptions of character and character traits portray a way of being instead of a way of acting… [A health worker] who responds to a difficult patient care situation with respect, patience and attitude of care is described as a “good” person ([18], p.49).

Aristotle’s philosophy and virtue-ethical assumptions have been interpreted in different ways, but all virtue theorists hold that the ability to make sound subjective judgments is a prerequisite for ‘good’ actions in patient work [19]. The idea has been that analyses of personal judgment are needed as alternatives to theories “characterized by a focus on right decisions and acts based on consideration of more abstract ethical principles” ([18], p.49).

This central assumption in virtue ethics has received much attention in health care ethics recent years [20–24]. Many have thought that if rigid rules become dominant in ethical guidelines and practical analyses, an important interpersonal dimension of professional health-care practice will be excluded. Thus, as Scott observes, “a number of contributors to the health care ethics literature have, for a number of years now, tried to argue that within the health care and nursing context, a virtue theory approach is needed at least as a supplement to a duty- and principle-based approach” ([20], p. 26).

The idea has been that a narrow focus on rules undermines health workers’ individual moral responsibility. Many have argued that rule-based ethics cannot take into account the clinical judgment, subjective commitment, responsibility and dedication necessary for consciously doing something good [17, 18]. The skill of being a competent practitioner is not equivalent to rule-following; it is impossible to state rules such that health workers necessarily display a genuine competence if the rules are followed [19, 20].

It is easy to understand how virtue ethics is relevant in the above case. It was the paramedics’ clinical judgment that saved the situation. Aristotle would say that they were ‘good’ professionals and did something ‘good’ because they used their skills as practitioners [19]. The paramedics had respect for the rules, but interpreted them contextually. Thus, the aim of developing relevant competence skills should not be understood as an aim of creating any kind of ‘relativistic’ attitudes – that everyone should be allowed to overrule procedures like DNR clauses if they think they have a personal good reason for doing so (see also above). On the contrary, the relevant competence should help health workers to uncover misunderstandings about factual matters that can lead to substantial mistakes. Such a capacity is not in any sense a ‘subjective’ competence that entitles everyone to place a parenthesis around guidelines and act in the way they think is correct. It should rather make it easier to conform to system-based values, like respecting patient wishes and *sound* DNR clauses.

The situation illustrates the important point that health workers need to find a balance. They need to learn technical procedures and corresponding technical skills, but they also need to use their own judgments when interpreting guidelines. Obviously, developing technical skills can be challenging, but it is normally reasonably clear what the aims are. Developing non-technical skills can be much more challenging. It is less clear what the pedagogical aims are and how they can be achieved. Nevertheless, they are crucial for being able to perform excellently in situations where lives are at stake [15, 16, 25].

## Conclusion

Developing personal judgment is a matter of developing non-technical skills. These skills are, as Fletcher [26] observes, “sometimes referred to under the general heading of ‘human factors’, but more specifically, as they do not relate directly to the use of medical expertise, drugs and equipment (i.e., clinical knowledge and technical skills), they can be described as non-technical skills”.

It is the technical skills, unfortunately, that get almost all attention in the pedagogical literature on competence requirements in emergency medicine [16]. For emergency personnel to develop excellent decision-making abilities, it is necessary to focus more on the non-technical skills. This is primarily a matter of developing communication skills. Paramedics need to learn principles such as ‘closing the loop’, which involves checking information, clarifying messages and following up on issues, actions or projects until they are successfully completed [2–4]. They also need to develop ethical integrity and acquire the capacity to make sound clinical judgments. As the case shows, this is especially important when the patient’s appearance suggests that a rule formulation does not lead to actions that are in the patient’s best interests. In order to ensure good treatment and avoid potentially negative consequences for patients in acute medical services, it is necessary to focus on all these abilities in the education and training of emergency personnel. As the case discussed in this article illustrates, the abilities can be of crucial importance in patient work.

## Consent

The participant in the advanced further education course for paramedics who provided narration to this article is aware of his contribution and has given informed consent to publication.

## Footnotes

1 The reason why it is necessary to use the qualification ‘normally’ is that it has been extensively documented that patients’ preferences and the role they have in end-of-life decision processes are shaped by cultural frameworks [27–30]. Furthermore, advanced directives are sometimes based on oral consent, for instance when an autonomous patient is not capable of reading or signing a document [5, 8]. However, the general practice of inserting DNR clauses in patients’ medical records have been implemented in public health services on a large scale, and are highlighted in international paramedics textbooks that are used in education and training of emergency personnel [11, 12].

2 A qualification similar to the point made in endnote 1 above applies. The role of relatives’ preferences depends on cultural frameworks [6, 27–29]. In some countries there is a strong tradition for involving relatives of patients in decision processes concerning the patient [27, 28]. In other countries (like Norway) relatives are only involved in end of life decision process if the patient is incapable of expressing autonomous beliefs. Discussing these cultural differences in detail fall outside the main focus of this article. My aim here is to make a general point about the importance of contextual interpretation of procedural directives.

3 There are no international standards for how the term ‘paramedic’ should be used. In some countries a paramedic needs to have a bachelor degree from university levels, in other countries this is not required. These differences are not crucial for the arguments here.

4 The specific formulations differ from area to area. For instance, the MOM in the capital area (Oslo) is not the same as in northern parts of Norway. The lack of national standards is unfortunate for many reasons, but focusing on this falls outside the main focus of this article.
